# Identification and assessment of novel dynamic biomarkers for monitoring non‐traumatic osteonecrosis of the femoral head staging

**DOI:** 10.1002/ctm2.1295

**Published:** 2023-06-14

**Authors:** Yan Jia, Yanqiong Zhang, Shuwen Li, Ruihan Li, Weijie Li, Taixian Li, Rongtian Wang, Guangrui Huang, Haijun He, Na Lin, Weiheng Chen

**Affiliations:** ^1^ Department of Minimally Invasive Arthrology Third Affiliated Hospital of Beijing University of Chinese Medicine Beijing China; ^2^ Research Center of TCM Theory and Materia Medica Literature Institute of Chinese Materia Medica China Academy of Chinese Medical Sciences Beijing China; ^3^ School of Life Sciences Beijing University of Chinese Medicine Beijing China; ^4^ The Third Department of Orthopaedics Wangjing Hospital of China Academy of Chinese Medical Sciences Beijing China

Dear Editor,

How to differentially diagnose non‐traumatic osteonecrosis of the femoral head (NONFH) patients during the transition from early to late stage accurately and identify the core regulators governing such a critical transition may be one of the clinical problems that urgently needed to be solved. Studies have revealed that the initiation and development of NONFH are intricately regulated by a transcriptional regulatory network.^1^, ^2^ To explore dynamic biomarkers to monitor NONFH staging, we herein divided NONFH patients into three stages according to radiological staging system, extensively collected their clinical symptoms, and formed a network fusion‐based analysis by combing the NONFH‐related expression profiling, clinical phenomics data and real‐time quantitative polymerase chain reaction (RT‐qPCR) validation (Figure 1).

**FIGURE 1:**
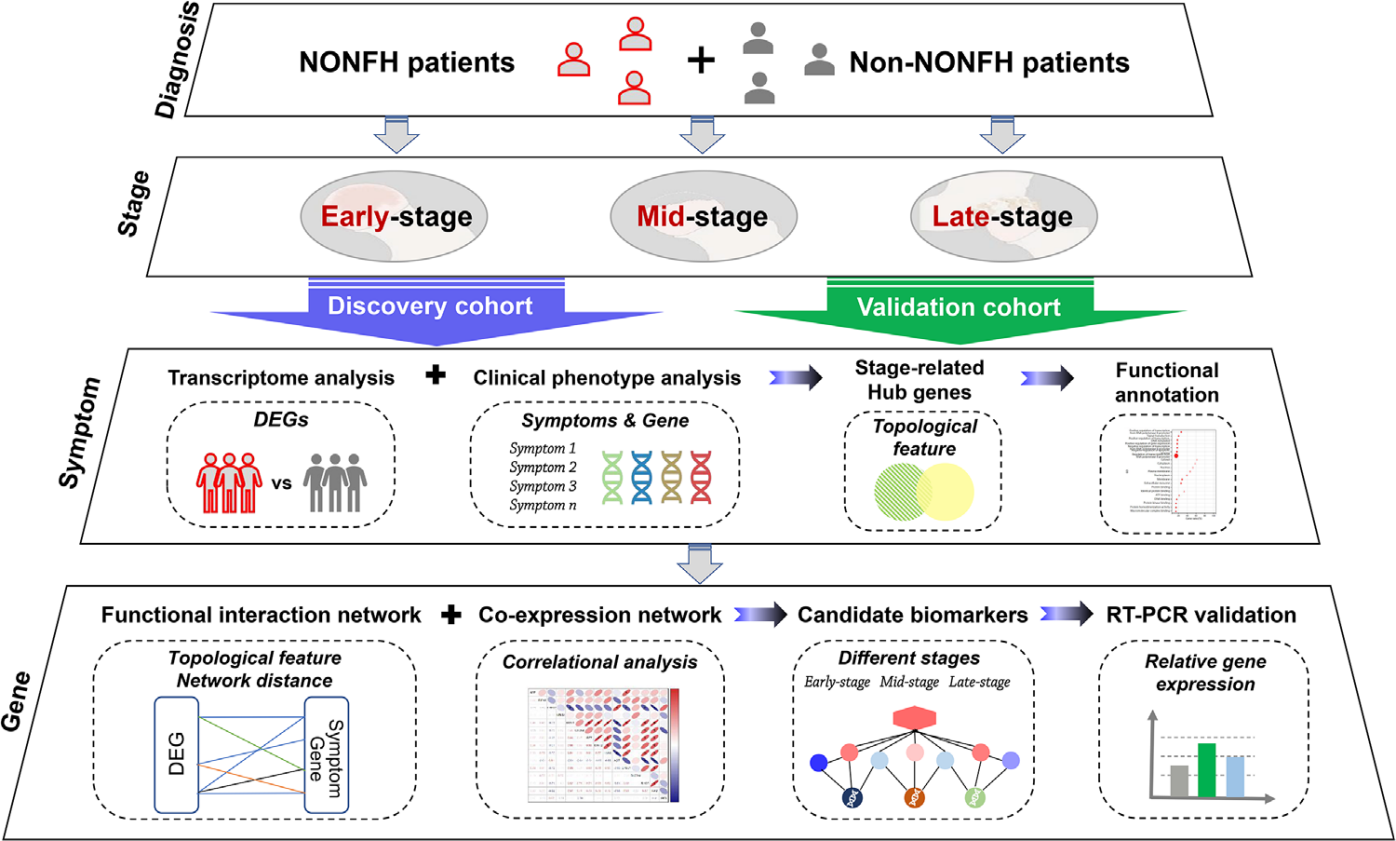
Schematic diagram of the systematic strategies to identify candidate biomarkers of non‐traumatic osteonecrosis of the femoral head (NONFH) at different stages.

A total of 84 steroid‐induced NONFH patients, who were staged into early‐, mid‐, and late‐stage groups based on the imaging system,^3^, ^4^ and 20 non‐NONFH patients were enrolled from January 2014 to August 2022, were randomly divided into a discovery cohort (10 per group) and a validation cohort (non‐NONFH control group = 10, early‐stage NONFH group = 22, mid‐stage NONFH group = 18, late‐stage NONFH group = 14) with strict primary disease of steroid inclusion criteria (Table S1). Differentially expressed genes (DEGs) obtained from peripheral blood samples of the discovery cohort (GEO accession: GSE123568) were identified by comparing gene expression profiling between each NONFH stage group and control group in our previous studies.^1^, ^2^ The hub DEGs of each NONFH stage group should be screened out through interaction network by using the public database Search Tool for the Retrieval of Interacting Genes (STRING, version 11.5, https://cn.string‐db.org) and Cytoscape v3.8.2 software to calculate the degree, betweenness centrality and closeness centrality. The typical clinical symptoms corresponding to different NONFH stages^5^, ^6^, ^7^ and the related genes were collected from Human Phenotype Ontology (HPO, released on June 12, 2022, https://hpo.jax.org/app), Disease‐Gene Association Network (DisGeNET, version 7.0, https://www.disgenet.org) and SoFDA database (http://www.tcmip.cn/Syndrome/front/#/).^8^ The disease‐related genes of each NONFH stage included hub DEGs and the typical clinical symptom‐related genes. To filter disease‐related hub genes, we constructed the disease‐related gene interaction network and set two screening criteria: a) PPI minimum required interaction score > 0.7 based on STRING database; b) higher than the median value of degree, betweenness centrality and closeness centrality calculated by Cytoscape v3.8.2 (Tables S2–5). Then, we distinguished symptom‐related genes from DEGs in disease‐related hub genes, calculated the percentage of disease‐related hub genes in each symptom‐related gene sets to reflect the intensity of associations between the typical clinical symptoms and hubs of NONFH at different stages, which were indicated as the participation rate. As a result, we found that the early‐disease‐related hubs were associated with 12 typical symptoms, among them, “episodic pain” had the highest participation rate of 42.11%, followed by “hip pain” (34.38%) and “epigastric pain” (32.86%) (Figure 2A). Regarding the mid‐disease‐related hubs, 12 typical symptoms were screened, among which “hip pain” (37.93%), “headache” (29.10%) and “ecchymosis” (24.14%) had higher participation rates (Figure 2D). In addition, the late‐disease‐related hubs were involved into 11 typical symptoms with a higher participation rate of “hip pain” (37.93%), “night sweats” (33.33%) and “low back pain” (32.18%) (Figure 2G). The functional enrichment analysis based on GO and KEGG indicated that the different stage‐related hubs may be involved into various biological processes and pathways related to the regulation of the immune system, signal transduction and main pathological changes during the progression of NONFH (Figure 2B,C,E,F,H,I).

**FIGURE 2:**
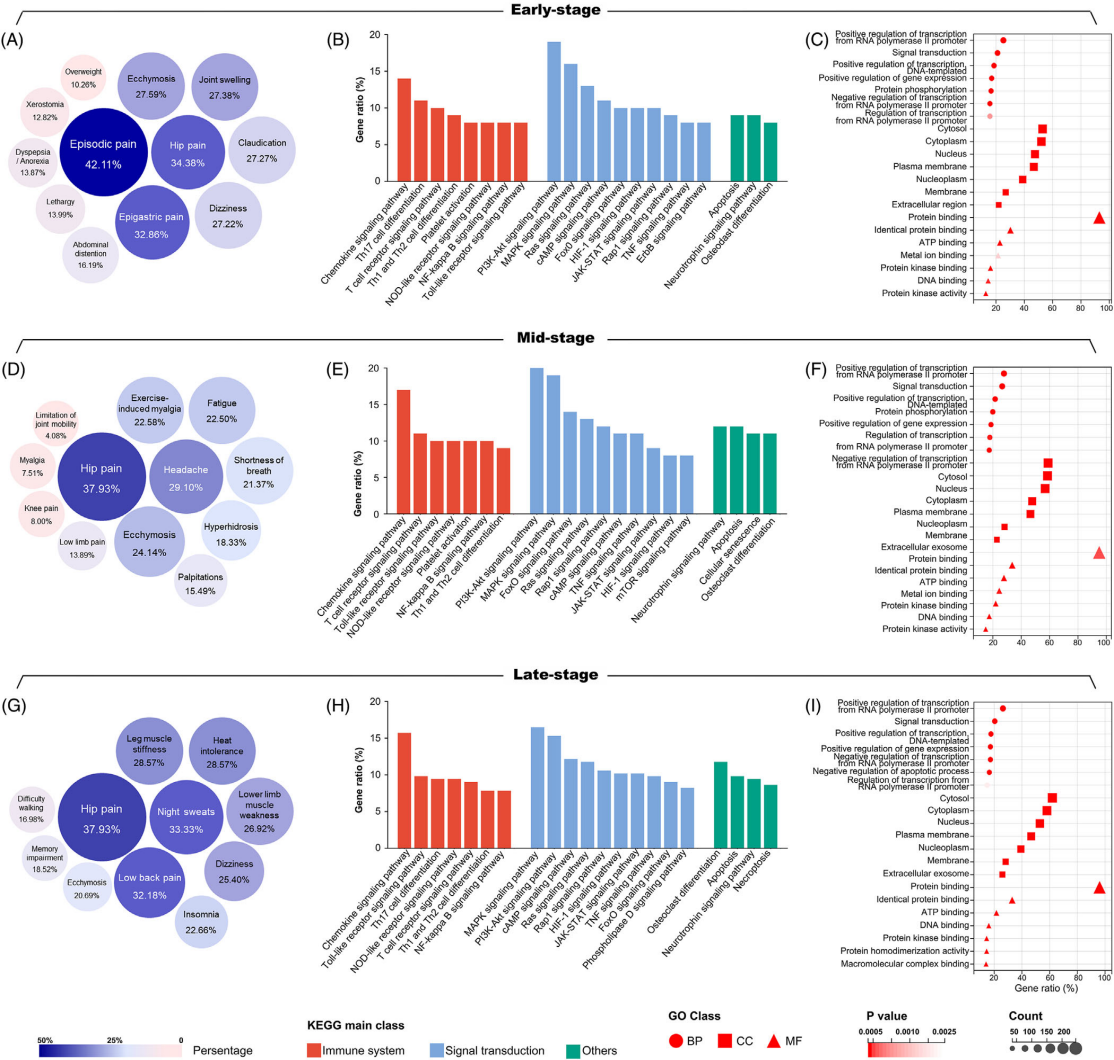
Disease‐related hubs participation rate and functional enrichment analysis of different non‐traumatic osteonecrosis of the femoral head (NONFH) stages. (A, D and G): The percentage of disease‐related hubs in symptom‐related genes of early‐stage, mid‐stage and late‐stage respectively. (B, E and H): The main 21 pathways of KEGG enrichment analysis related to NONFH at early‐stage, mid‐stage and late‐stage, respectively. (C, F and I): Top 20 function terms of GO analysis of early‐stage, mid‐stage and late‐stage, respectively; BP, biological process; CC, cellular component; MF, molecular function.

The non‐overlapping hub genes of NONFH at each stage were selected for further analysis (Figure 3A–G). The corresponding fold change (FC), *p*‐value, and expression patterns were collected, and their network distance to the corresponding symptom‐related genes was evaluated by the average shortest path length. The correlation analysis was also carried out to evaluate the expression relevance among the hub genes. To identify candidate biomarkers of different NONFH stages, four screening criteria were further set as follows: 1) average shortest path length < median; 2) co‐expression correlation *p*‐value < 0.05; 3) log2 FC > median; 4) differentially expressed *p*‐value < median. Any three out of four criteria were sufficient (Tables S6–8). As a result (Figure 3H and Figure S1), we identified correlative 6 genes for early‐stage (PAK2, CD28, CD4, PIK3CD, PLCG1 and PRKCA), 13 genes for mid‐stage (VCL, IQGAP1, ACTN4, RAC1, XIAP, PIK3CB, TRAF6, PSEN1, TUBA1A, YWHAZ, HDAC4, CSNK1D and STK11) and six genes for late‐stage (STAT2, STAT1, ERBB2, CXCR4, AGT and LPAR1).

**FIGURE 3:**
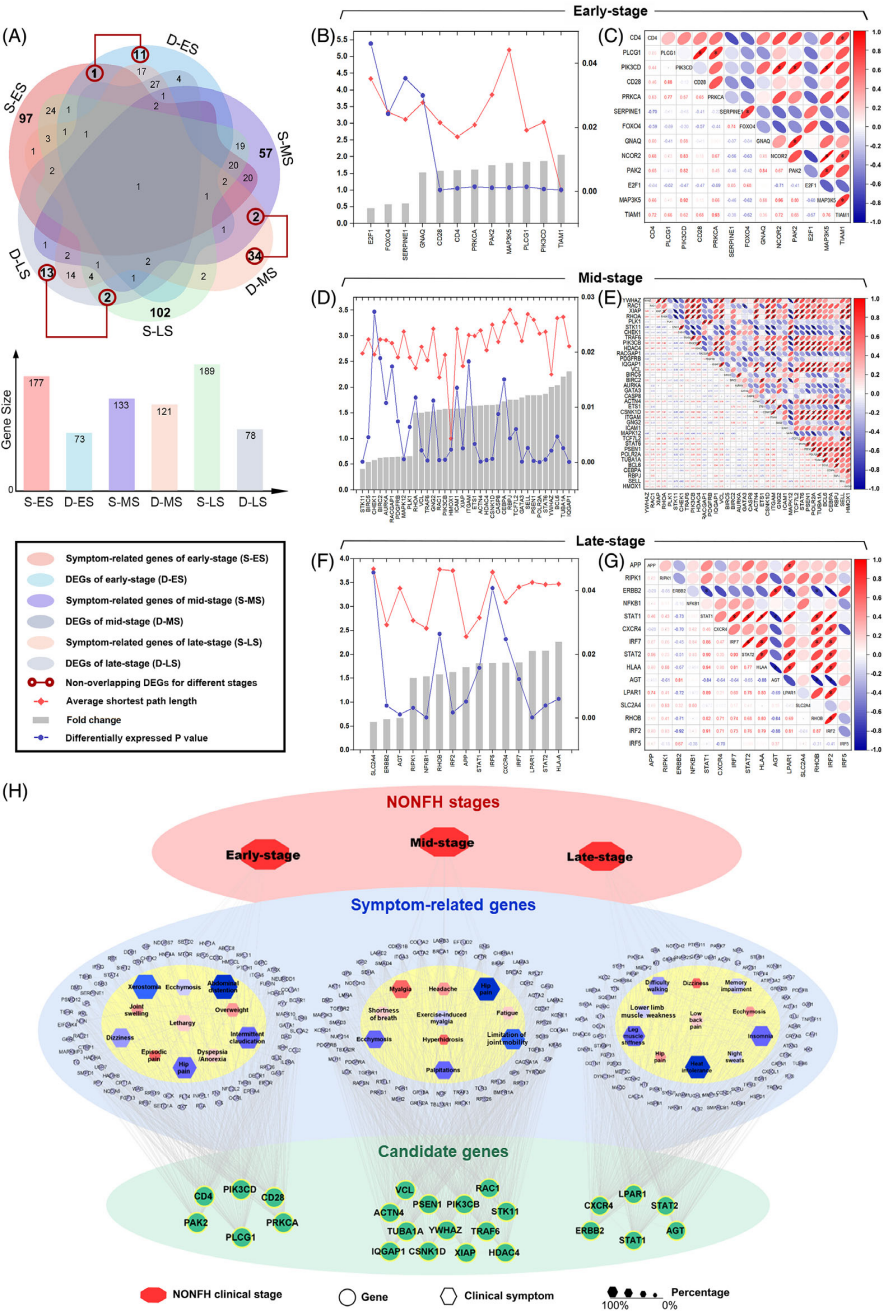
The disease‐related hubs interaction networks of different non‐traumatic osteonecrosis of the femoral head (NONFH) stage. (A) Venn diagram of NONFH stage‐related hubs consisting of symptom‐related genes and differentially expressed genes (DEGs). The non‐overlapping DEGs between three NONFH stage‐related hubs were 12, 36 and 15, respectively. (B) The network comparison of the early‐stage. Median (average shortest path length) = 3.299; median (fold change) = 1.604; median (*p‐*value) = 0.001. (C) The co‐expression correlation analysis of the early‐stage. *p < 0.05. (D) The network comparison of the mid‐stage. Median (average shortest path length) = 3.109; median (fold change) = 1.605; median (*p‐*value) = 0.003. (E) The co‐expression correlation analysis of the mid‐stage. (F) The network comparison of the late‐stage. Median (average shortest path length) = 3.545; median (fold change) = 1.733; median (*p‐*value) = 0.005. (G) The co‐expression correlation analysis of the late‐stage. (H) Candidate biomarkers were associated with clinical stage through typical symptoms and symptom‐related genes respectively. The clinical symptom with a large area indicated participation was higher.

RT‐qPCR was detected using the peripheral blood RNA samples obtained from the validation cohort to further verify the expression patterns of candidate biomarkers for different NONFH stages (Table S9–11). The relative expression levels of PRKCA, PIK3CD, CD4, IQGAP1, TUBA1A, VCL, STAT2, CXCR4 and LPAR1 mRNAs were consistent with those of transcriptomics data (Figure 4A,D,G). Among them, the relative expression levels of PRKCA, PIK3CD and CD4 mRNA were all dramatically up‐regulated in the NONFH early‐stage patients compared with the non‐NONFH control group (NC group) and other stage groups (N&S groups) (all *p* < 0.05, Figure 4A). IQGAP1, TUBA1A and VCL mRNA expression levels were increased in the mid‐stage NONFH patients compared with the NC group, and TUBA1A had significant differences from N&S groups (*p* < 0.05, Figure 4D). STAT2, CXCR4 and LPAR1 were all highly expressed in the late‐stage NONFH patients (all *p* < 0.05, Figure 4G). As indicated from the receiver operating characteristic curve, PRKCA, PIK3CD and CD4 had a good performance for the early‐stage NONFH patients compared with the NC group and other disease groups as well (all *p* < 0.05, Figure 4B). STAT2, CXCR4 and LPAR1 displayed a predictive performance for the late‐stage NONFH patients (all *p* < 0.05, Figure 4H). However, only TUBA1A expression showed a significant difference between mid‐stage NONFH patients and controls (*p* < 0.01, Figure 4E). Furthermore, the accuracy of those genes all reached 50%, with CD4 being the most preferred at 88% for early‐stage patients and STAT2 at 85.71% for late‐stage patients. The precision was also good for early‐ and late‐stage patients, as CD4 was the highest at 100%, followed by PRKCA (94.12%) and STAT2 (85.71%), and so was their F1 measure trend. (Figure 4C,F,I).

**FIGURE 4:**
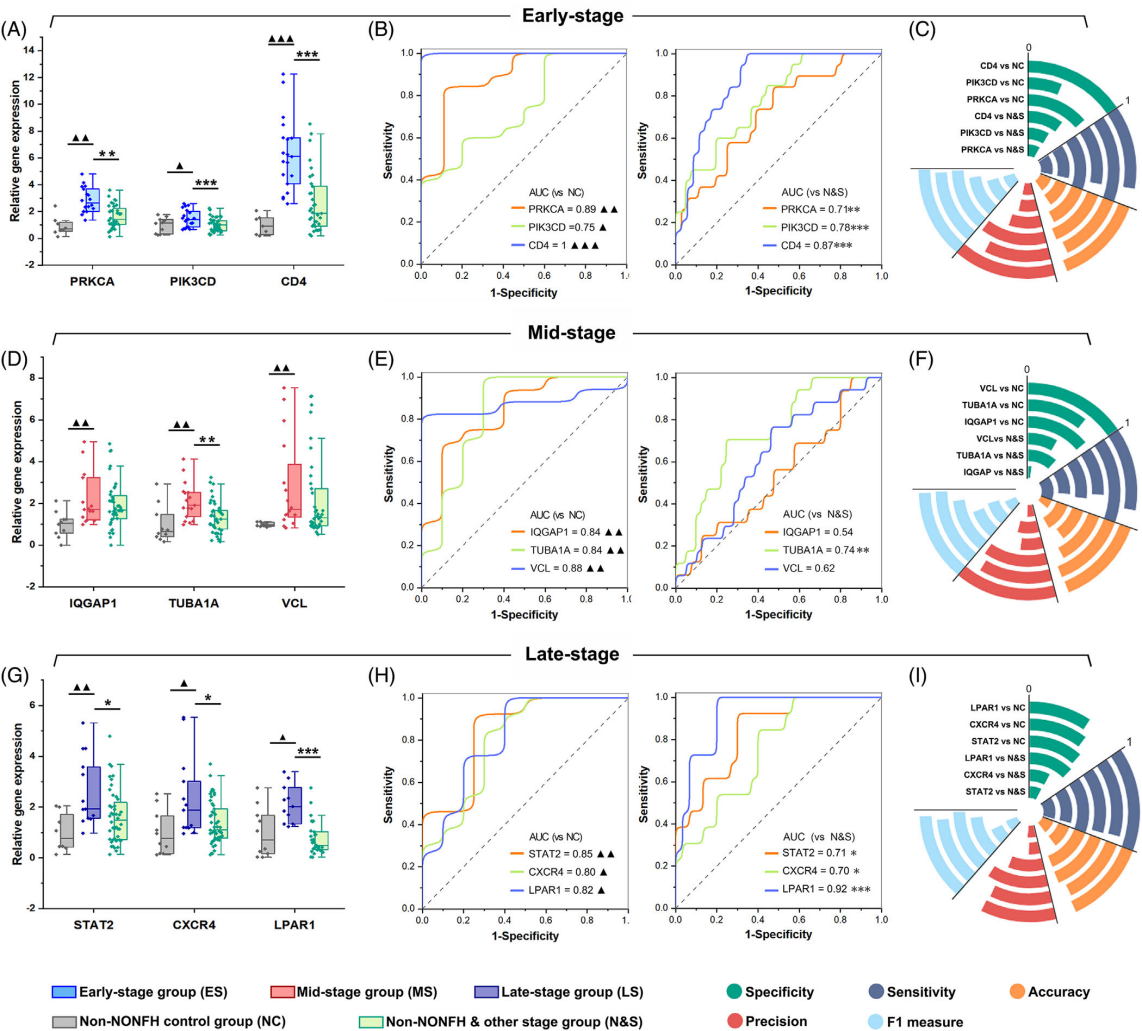
Expression levels of candidate gene biomarkers and receiver operating characteristic (ROC) curve analysis. (A, D and G): Relative gene expression of candidate genes via real‐time polymerase chain reaction (RT‐PCR) between non‐traumatic osteonecrosis of the femoral head (NONFH) patients and non‐NONFH patients. For early‐stage, the N&S group consisted of the non‐NONFH control group, mid‐stage group, and late‐stage group; for mid‐stage, the N&S group consisted of the non‐NONFH control group, early‐stage group, and late‐stage group; for the late‐stage group, the N&S group consisted of the non‐NONFH control group, early‐stage group, and mid‐stage group. (B, E and H): AUC comparison on different groups of candidate gene biomarkers. (C, F and I) Prediction features of NONFH candidate gene biomarkers. The range of specificity, sensitivity, accuracy, precision and F1 measure is 0–1. ▲/*, *p*<0.05; ▲▲/**, *p*<0.01; ▲▲▲/***, *p*<0.001, compared with NC group and N&S group, respectively.

In conclusion, six promising genes (PRKCA, PIK3CD and CD4 for the early stage; STAT2, CXCR4 and LPAR1 for the late stage) among a list of novel candidate dynamic biomarkers to monitor NONFH staging were identified through the integrative network fusion‐based transcriptomics and clinical phenomics data. Among them, PRKCA and PIK3CD may regulate cell proliferation, differentiation, and immunoreaction with CD4, which has been reported to be involved in bone metabolism and blood circulation^9^; STAT2, CXCR4 and LPAR1 may control bone resorption by regulating the differentiation or movement of osteoclast and osteoblast, and CXCR4 may be also associated with angiogenesis.^10^ Our study extends the exploration strategy of accurate disease prediction and may be of significance to improve NONFH diagnosis and therapy.

## CONFLICT OF INTEREST STATEMENT

The authors declare no conflict of interest.

## Supporting information

Supporting Information
