# Supplementary material for: Identification and assessment of novel dynamic biomarkers for monitoring non‐traumatic osteonecrosis of the femoral head staging
Source: Clin Transl Med. 2023 Jun 14;13(6):e1295. doi: 10.1002/ctm2.1295 (PMC10265435; doi:10.1002/ctm2.1295)
Supplement: Supplementary file 1 — Supporting Information [file CTM2-13-e1295-s001.docx]

**Supplement**

**Statement of Ethics**

Ethics was approved by the research ethics committee of institute of Wangjing Hospital of China Academy of Chinese Medical Sciences (SFDA approval number: 81473695) and Third Affiliated Hospital of Beijing University of Chinese Medicine (BZYSY-2021KYKTPJ-01). Informed consent was obtained from all participants included in the study.

**Funding**

This study was supported by National Natural Science Foundation of China (No.82030122; No. 81973888), Beijing University of Chinese Medicine High-level Talent Research Startup Project (2021-XJ-KYQD-001).

Table S1 Clinical summary of study subjects

|  | **Early-stage group** | **Mid-stage group** | **Late-stage group** | **Control group** | ***P*** |
| --- | --- | --- | --- | --- | --- |
| ARCO stage | ARCO Ⅰ-Ⅱ | ARCO Ⅱ, Ⅲ (A) | ARCO Ⅲ (B), Ⅳ | / | */* |
| Imaging feature | Cystic changes, focal bone mineral density reduction | Subchondral fracture, focal radiolucency | Severe femoral head depression, osteoarthritis | Normal | / |
| Primary affection of steroid | Dermatosis; systemic lupus erythematosus; nephropathy; hemopathy; acute inflammation | Nephropathy; hemopathy; dermatosis; systemic lupus erythematosus; facial paralysis;tinnitus | Systemic lupus erythematosus; nephropathy; rheumatism; rhinitis; autoimmune hepatitis | Hemopathy | / |
| Discovery cohort |  |  |  |  |  |
| Gender  Male  Female | 6  4 | 6  4 | 1  9 | 7  3 | 0.032 |
| Age(mean±SD) | 43.10±7.64 | 48.60±13.67 | 31.80±10.03 | 38.80±10.86 | 0.010 |
| BMI (kg/m^2^) | 24.03±2.96 | 24.14±2.02 | 20.80±1.92 | 25.02±3.01 | 0.003 |
| During of steroid (month) | 13.50±12.39 | 19.50±8.09 | 43.20±25.93 | 28.30±36.04 | 0.052 |
| Validation cohort |  |  |  |  |  |
| Gender  Male  Female | 12  10 | 13  5 | 2  12 | 5  5 | 0.01 |
| Age(mean±SD) | 43.64±11.39 | 39.33±17.39 | 38.86±14.72 | 34.00±15.54 | 0.376 |
| BMI (kg/m^2^) | 25.49±3.81 | 23.49±3.44 | 23.51±3.19 * | 24.21±2.04 | 0.241 |
| During of steroid (month) | 28.19±38.50 | 15.03±13.32 | 35.14±30.12 | 17.20±3.74 | 0.198 |

* *P*<0.05, compared with the same stage patients in discovery cohort.

Table S2 Summary of different gene sets

| Stage | Typical Symptom Number | Symptom-related Genes | Hub DEGs | Stage-related hub  (Symptom gene/DEG) |
| --- | --- | --- | --- | --- |
| Early-stage | 15 | 909 | 101 | 245  (177/73) |
| Mid-stage | 14 | 749 | 154 | 242  (133/121) |
| Late-stage | 11 | 853 | 101 | 257  (189/78) |

Table S3 Hub DEGs of different NONFH stages

| Stage | DEGs |
| --- | --- |
| Early-stage | ADCY7,AKT1,AKT2,ARHGDIA,ATM,BCL2,BCL2L1,BIRC2,BIRC3,CBL,CCR5,CD28,CD36,CD4,CDC25A,CDK1,CDKN1B,CEBPB,CREB1,CREBBP,CSK,E2F1,ETS1,FGR,FOXO1,FOXO3,FOXO4,GNAQ,GRB2,GSK3B,HCK,ICAM1,IGF1R,IKBKB,IRS2,ITGAX,JAK1,JAK3,KAT2B,LAT,LCK,LPAR1,LPAR2,LYN,MAP2K4,MAP3K1,MAP3K5,MAPK1,MCL1,MYD88,NCOA1,NCOA3,NCOR2,NOTCH1,PAK1,PAK2,PDPK1,PIK3CD,PIK3R5,PLCG1,PPM1F,PRKACA,PRKAR1A,PRKCA,PRKCB,PRKCD,PRKCZ,PRKDC,PTAFR,PTEN,PTGS2,PTPRC,PXN,RAC2,RAF1,RALBP1,RHOG,RHOQ,RPS6KA1,RPS6KA3,RUNX2,S1PR1,S1PR3,SERPINE1,SH3KBP1,SKP1,SP1,STAT3,STAT5A,STAT5B,STK11,SYK,TFDP1,TIAM1,TLR2,TLR4,TNFRSF1A,TSC1,TYK2,YWHAB,ZAP70 |
| Mid-stage | ACTN4,ADCY7,AKAP13,AKT1,AKT2,ARRB1,ATM,AURKA,BCL2L1,BCL6,BIRC2,BIRC3,BIRC5,CASP8,CBL,CCR5,CD36,CDC20,CDC25A,CDC27,CDK1,CDK8,CDKN1B,CEBPA,CEBPB,CFLAR,CHEK1,CREB1,CREBBP,CRK,CSF1R,CSK,CSNK1D,CUL1,CXCL12,DNM1,DYNC1H1,ETS1,FGR,FOS,FOXO1,FOXO3,FOXO4,GATA3,GNAI3,GNG2,GRB2,GSK3B,HCK,HDAC4,HMOX1,ICAM1,IGF1R,IKBKB,IL1B,IL8,IQGAP1,IRF1,IRS2,ITGA2,ITGAM,ITGAX,JAK1,JAK2,JAK3,KAT2B,LCK,LRRK2,LYN,MAP2K1,MAP3K1,MAP3K3,MAP3K5,MAP3K7,MAPK1,MAPK12,MAPK14,MAPK3,MBP,MCL1,MEF2D,MSN,MYD88,NCF4,NFKBIA,NOTCH1,NOTCH2,NR3C1,PAK1,PDGFRB,PDPK1,PIK3CB,PIK3CD,PLK1,POLR2A,PPM1A,PPM1F,PRKACA,PRKAR1A,PRKCB,PRKCD，PRKCZ，PRKDC，PSEN1，PTEN，PTGS2，PTPRC，PXN，RAC1，RAC2，RACGAP1，RAF1，RAP1A，RBPJ，RBX1，RHOA，RHOB，RHOG，RPS27A，RPS6KA1，RPS6KA3，RPS6KA5，RUNX2，RUNX3，SELL，SGK1，SKP1，SLC2A4，SNCA，SOS2，SP1，STAT2，STAT3，STAT5A，STAT5B，STAT6，STK11，SYK，TBL1XR1，TCF7L2，TLR2，TLR4，TRAF6，TSC1，TUBA1A，TUBB4B，TUBG1，TYK2，UBE2D1，VAV1，VCL，XIAP，YWHAB，YWHAZ |
| Late-stage | AKT1,ARRB1,BCL2L1,CBL,CDKN1B,CREB1,CXCR4,DNM1,FOS,GSK3B,IL1B,ISG15,LPAR1,MAPK1,NFKB1,NFKBIA,PDPK1,PRKACA,PRKCD,PTEN,SLC2A4,STAT3,UBE2D1,AGT,AKT2,APP,ARHGEF12,BIRC3,CCR5,CCR7,CDK8,CEBPB,CREBBP,CRK,CUL1,CXCL1,CXCL12,CXCR2,EP300,ERBB2,ETS1,FGR,FOXO1,FOXO3,GNAI3,GRB2,HCK,HLA-A,IFIT1,IL8,IRF1,IRF2,IRF5,IRF7,IRF9,ITSN1,JAK1,JAK2,JAK3,KAT2B,LCK,LPAR2,LYN,MAP2K1,MAP2K4,MAP3K1,MAP3K7,MAPK14,MYD88,NCOA1,NCOA3,NOTCH1,PAK1,PIK3CD,PRKCZ,PTAFR,PXN,RAC2,RACGAP1,RAF1,RALBP1,RBX1,RHOB,RHOG,RHOQ,RHOU,RIPK1,RPS27A,S1PR1,SKP1,SP1,STAT1,STAT2,STAT5A,STAT5B,SYK,TLR4,TNFRSF1A,TYK2,VAV1,YWHAB |

Table S4 Clinical symptom collection of different NONFH stages

| Stage | Symptom Term Name |
| --- | --- |
| Early-stage | Hip pain/Arthralgia of the hip; Episodic pain/Paroxysmal pain; Joint swelling; Intermittent claudication; Chest tightness; Obesity/ Overweight; Anorexia/Dyspepsia; Vertigo/Dizziness; Xerostomia; Epigastric pain; Abdominal distention; Loose stool; Lethargy; Ecchymosis; Tongue edema |
| Mid-stage | Hip pain/Arthralgia of the hip; Knee pain; Low Limb pain; Exercise-induced myalgia; Limitation of joint mobility; Muscle tenderness; Fatigue; Myalgia; Generalized morning stiffness; Palpitations; Headache; Hyperhidrosis; Shortness of breath; Ecchymosis |
| Late-stage | Hip pain/Arthralgia of the hip; Leg muscle stiffness; Lower limb muscle weakness; Difficulty walking; Low back pain; Vertigo/Dizziness; Memory impairment; Insomnia/ Sleeplessness/ Sleep onset insomnia /Difficulty sleeping; Night sweats; Heat intolerance; Ecchymosis |

Table S5 Symptom genes collection

| Symptom | Term identifier | Genes |
| --- | --- | --- |
| Hip pain/  Arthralgia of the hip | [HP: 0030838](https://hpo.jax.org/app/browse/term/HP:0030838),  [0003365](https://hpo.jax.org/app/browse/term/HP:0003365);  UMLS: [C4551516](https://www.disgenet.org/browser/0/1/0/C4551516/) | BSCL2,COL2A1,HNRNPA1,HNRNPA2B1,LMNA,MATN3,TRPV4,VCP, ZMPSTE24,DMD,FIP1L1,BEST1,AURKB,CCL22,RHAG,PDGFRA,SLC45A2,ACR,ATF3,ADAM11,IFNG,CRYBG1,COMP,AIM2,B2M,COL9A1,COL9A2,COL9A3,NGF |
| Episodic pain | HP: [0032148](https://hpo.jax.org/app/browse/term/HP:0032148);  UMLS:C3808667, C3809893, C3809899 | ATRX,CDKN1B,CPT2,DNMT3A,ESR1,LPL,MAX,NF1,RET,CDC73,DLST,EPAS1,F12,FH,FLI1,GNE,HEXB,KIF1B,MC2R,MDH2,MEFV,MRAP,NNT,SDHA,SDHAF2,SDHB,SDHC,SDHD,SLC25A11,STAR,TGFBI,TMEM127,TXNRD2,VHL,SCN10A,SCN11A,TRPA1,MSC-AS1 |
| Joint swelling | [HP: 0001386](https://hpo.jax.org/app/browse/term/HP:0001386);  UMLS: C0152031 | ACTC1,ALB,CITED2,FLT4,FN1,GATA4,MYD88,NKX2-5,PRKAR1A,CD247,CHEK2,COL1A1,COL5A1,COL5A2,FGA,FGB,FGG,HLA-DRB1,IL10,IL2RA,IL2RB,IL6,RB1,STAT4,TP53,PTPN2,APC2,EIF2AK4,GATA6,GBA,GJC2,KIF1A,MAGEL2,MEFV,MKKS,MYH6,NDN,NSD1,OCA2,PLAA,RBM8A,RNF13,SCARB2,SETD2,SNORD115-1,SNORD116-1,SNRPN,TBX20,TET2,TGM1,TLL1,VEGFC,ZNHIT3,ADM,ACP5,AIP,ANKH,ASAH1,ATP7B,BTNL2,CCN6,CD244,CIITA,DDR2,F8,GPR101,HFE,HGD,HPGD,IL1RN,LACC1,LMX1B,LPIN2,MIF,NFKBIL1,NOD2,OCRL,OTULIN,PTPN22,SLC22A4,SLC40A1,SLCO2A1,TNFRSF11B,ANKRD55 |
| Tongue Edema | [HP: 0040315](https://hpo.jax.org/app/browse/term/HP:0040315) | SERPING1 |
| Ecchymosis | [HP: 0031364](https://hpo.jax.org/app/browse/term/HP:0031364);  UMLS: C0013491 | NABP1,F13B,GGCX,NUMA1,IRF2BP2,CHST14,GFI1B,TBXAS1,SBDS,TBXA2R,GP6,CD109,USP8,FIP1L1,F13A1,GP1BA,ZBTB16,PRF1,TBL1XR1,P2RY12,GP1BB,ITGA2,AIP,MPL,COL1A1,COL3A1,COL5A1,COL5A2,GBA |
| Chest tightness | [HP:0031352](https://hpo.jax.org/app/browse/term/HP:0031352);  UMLS: [C0232292](https://www.disgenet.org/browser/0/1/0/C0232292/) | COPD |
| Obesity/ Overweight | HP:0025502, 000151  UMLS:C0028754, C0497406 | ABCB4,ARMC5,BAP1,BLM,BPTF,CDH23,CEP164,CFAP418,COA3,COL10A1,COPB1,DDB1,DDX6,DNAJC30,DYNC2I2,DYRK1B,EXOC6B,FMR1,FXR1,GNAS-AS1,HDAC8,HERC2,IPW,LARS2,MAN1B1,MAPK8IP3,MCM3AP,MIA3,MKRN3,MKRN3-AS1,MTFMT,NDNF,NIPBL,NKAP,NPAP1,OCA2,P4HTM,PCNT,PHF21A,PIGT,PKDCC,PSMD12,PWAR1,PWRN1,RAD21,RNPC3,RP1L1,SATB1,SETD1A,SLC10A7,SLC25A4,SLC7A7,SMARCE1,SMC1A,SMC3,SNORD115-1,SNORD116-1,SPEN,STEEP1,TCF20,TERT,THOC2,TOGARAM1,TRAF3IP1,TRAF7,TRIP12,WAC,XRCC4,XYLT1,ZBTB20,ZPR1,COX7C,ZFR2,PLSCR3,GHRLOS,BCDIN3D-AS1,CCDC77,LCE1C,FAM71F1,ZSCAN30,WAGRO,MIR4777,MIR4460,MIR4517,MACORIS,HAUS1,CCDC171,CFAP61,CCDC57,FLJ33534,EEF1A1P11,TMEM160,PLAAT2,RPL7P9,FN3KRP,PERM1,ZNF169,GPR12,LRRC53,LRRN4,EML6,AP3S2,LINC00689,LINC01412,LINC00237,ABHD15,ZNF543,GPAT4,ATP8B3,PHETA2,GEMIN5,ZZZ3,GM2AP1,HID1,DNAJB3,GPCPD1,SEMA3G,MCHR2-AS1,POLR1B,SCRN2,ZDHHC24,AP4S1,AP4E1,ACADVL,PAX4,TRIP4,AP4M1,AP4B1,C8orf37,KLF11,APPL1,IGSF1,BBS1,BLK,PPP3CA,NEUROD1,PDX1,KCNJ11,GCK,CEL,ABCC8,CHD8,FOXP1,HESX1,HNF1A,HNF4A,INS,LHX3,LHX4,POU1F1,PROP1,SPG11,SPTBN1,TRHR,UNC45B,USP7 |
| Anorexia/  Dyspepsia | [HP:0002039](https://hpo.jax.org/app/browse/term/HP:0002039), [0410281](https://hpo.jax.org/app/browse/term/HP:0410281);  UMLS:C0003123, C0013395 | ASXL1,CBL,CDKN1B,MEN1,PKHD1,RUNX1,GBA,HCRT,PTGDS,MPL,MBP,DBH,ZGLP1,STAR,NPPB,CRHR1,GHRL,UMOD,TNFRSF10A,ND1,SLC4A1,NTRK3,MIR23B,GHSR,IFNGR1,ND5,CCR1,TRNL1,MMUT,TBL1XR1,MC4R,GLA,ACAT1,CD247,MNAT1,NUCB2,PTGES,ARVCF,OXTR,PAM,ND6,DAXX,ZBTB16,ND4,COX3,ALPL,TXNRD2,HTR1B,TRNS1,RMC1,ERAP1,HMBS,MAGEL2,MIR340,PRSS1,TRNW,C4A,NTSR1,AVPR2,GIP,RCBTB1,GRHL3,ARC,FIP1L1,CARTPT,TRNS2,AQP2,TRNF,MC2R,OR2AG1,CRHR2,SLC5A1,CCL28,PYY,TRNQ,GOLGA6A,UCN3,TRNH,NNT,NFAT5,SCARB2,SLC46A1,SHCBP1,MAPK10,NPSR1,PLCL1,HTR4,NMU,CDAN1,NPY2R,C1QL1,CD3E,CCKAR,OXT,UCP3,CD200R1,CD3D,ECT,OPRL1,NPY1R,ABAT,SLC39A4,PALLD,OGN,UCN2,IL12A-AS1,SLC19A2,UBAC2,IRF2BP2,LRIT1,NUMA1,MMACHC,PTPN3,NCOA6,HTR2B,HLCS,CTRC,MLN,PDE3B,KLRC4,MRAP,HTR3B,HMGCL,CARD16,MC3R,HCRTR2,NABP1,MLX,NUCB1,IK,DYRK3,MMADHC,NMUR2,INSL5,GPR88,PYY3,GFRAL,KCNJ11,COL4A5,COL4A6,VMA21,PRLHR,MIR325,SYMPK,CELA3B,PPY,SSTR3,CYSLTR2,KCNIP3,MCM5,HTR3A,UGT1A9,ATP12A,ATP4A,AGA,SCN10A,SEMA4D,CTBP1,TPH1,CYBA,GNB3,GGTLC4P,GGTLC5P,GGT2,GGTLC3,SLC26A4,SERPINB2,FAP,GGT1,CHGA,GAST,IL17F,CYP2C19,PTGS1,TRPV1,FHIT,CHD7,CRH,S100A8,SLC6A4,DECR1,MIF,SLCO6A1,GSTK1,HP,HLA-DQB1,COMT,CD14,GABPA,IL1RN,FOXP3,SERPINE1,NFE2L2,IL17A,PTGS2,TGFB1,IL1B,TP53,TNF,AChE,CCKBR,GLP1R,MLNR |
| Vertigo/  Dizziness | [HP:0002321](https://hpo.jax.org/app/browse/term/HP:0002321)  UMLS:C0042571, C0012833 | H1R,GRXCR1,TECRL,TPK1,STRN4,CKMT1B,GYG1,CHRNA6,NABP1,CHRNB3,STRC,SPRTN,TRDN,CASQ2,GCDH,PFN2,TUBB6,FOXI1,KLRC4,ASIC3,NOP56,KCNJ13,COCH,HRH4,CACNB4,NAGA,CHRNB4,NUMA1,IRF2BP2,CNNM2,CLCN1,SGSH,UBAC2,TRPM4,IL12A-AS1,JUP,TMEM94,HINT1,CHRNB2,ADCY5,SLC2A4RG,TES,MYL2,KCNJ10,ATP1A2,PRIMA1,FES,CLCNKB,CACNA1G,CHRNA3,AFG3L2,CCT,MYO7A,CRY1,SIL1,MVK,SCN1B,FIP1L1,SLC1A3,KCNA1,ERF,LGI1,SLC25A4,SPAG9,C4A,GFER,PANK2,DSPP,ERAP1,ADRB1,RABGEF1,TERF2IP,ADA2,ZBTB16,RYR2,CDH23,GLA,TBL1XR1,NKX2-5,CHRNA7,CCR1,SH2B3,EPOR,GRM5,SCN2A,AIP,SCN5A,FKRP,THPO,ZGLP1,CYP3A5,DBH,LRRK2,MPL,SLC26A4,CCHCR1,NPPA,RAP1A,ARHGEF2,CYP3A4,CASP3,TNF,SLC22A2,IL1B,IL6,BCL2,ABCB1,FOS,CAT,MAPK1,MAPK3,CYP2D6,BAX,KCNH2,BDNF,IL10,REN,NOS2,RELA,VEGFA,PRL,IFNG,CCL2,CXCL8,BRV2,BPPV |
| Xerostomia | [HP:0000217](https://hpo.jax.org/app/browse/term/HP:0000217);  UMLS: C0043352 | ASXL1,CBL,MAGEL2,RUNX1,SLC12A2,TET2,ANG,TBK1,TREM2,HNRNPA1,FGF10,SCN9A,CHMP2B,OPTN,FIG4,PFN1,PON2,EPHA4,NEK1,NEFH,AFG3L2,DCTN1,PRPH,CHCHD10,CLDN10,TAF15,GLE1,MATR3,CFAP410,DAO,EDARADD,ANXA11,UBQLN2,PON3,VAPB,TUBB6,CCNF,UNC13A,GLT8D1 |
| Epigastric pain | [HP:0410019](https://hpo.jax.org/app/browse/term/HP:0410019);  UMLS: C0232493 | CFI,ALMS1 |
| Abdominal distention | [HP:0003270](https://hpo.jax.org/app/browse/term/HP:0003270) | BMPER,COL11A1,COL11A2,CYP27B1,CYP2R1,DYNC2I1,DYNC2I2,EPCAM,G6PC1,GBA,GNE,GNPTAB,HMBS,IFT122,IFT52,INPPL1,KAT6A,MTOR,NKX3-2,PLEC,PSMB8,PTH1R,PYGL,SI,SLC37A4,SMARCAL1,SMPD1,VDR,ACTG2,IRAK1,SLC26A4,MYLK,KRT8,SLC4A1,EPHB4,WDR35,ITGB4,RRM2B,MYH11,WAS,KRT18,TNFSF15,PSAP,NKX2-5,DUOX2,FSHR,ITGA6,ANK1,POU1F1,DLL3,SLC26A2,THRB,SLC26A3,DYNC2H1,MNX1,IL12RB1,SPINT2,POLG2,G6PC,HESX1,LIPA,THRA,FREM2,SLC2A2,FOXE1,IFT80,PROP1,TCTN2,SLC5A1,WDR60,TRIP11,WDR34,NPHS1,INHBA,MMEL1,POU2AF1,PIGQ,SLC9A3,LHX4,C1QA,ALDOB,UNC80,SPIB,SPTA1,C1R,TTC7A,MYO5B,LHX3,LMOD1,SPTB,MESP2,ALG8,TNPO3,TSHB,PHKG2,PRKCSH,PHKA2,SAR1B,DUOXA2,IYD,TRMU,EPB42,DDRGK1,SEC63,STX3 |
| Loose stool | UMLS: C2129214 | CORT, ADGRV1,TPH2,TFF3 |
| Lethargy | [HP:0001254](https://hpo.jax.org/app/browse/term/HP:0001254);  UMLS: C0023380 | ADA2,ATP5MK,CDKN1B,CDON,COX5A,CYTB,DDC,DLL1,DUOX2,DUOXA2,FGF8,FGFR1,GATA1,GLI2,HESX1,IYD,LHX3,LHX4,MAGEL2,MTHFR,NDUFAF8,NFS1,NODAL,PIGA,POU1F1,PROP1,PTCH1,RPL11,RPL15,RPL18,RPL26,RPL27,RPL35,RPL35A,RPL5,RPS10,RPS15A,RPS17,RPS19,RPS24,RPS26,RPS27,RPS28,RPS29,RPS7,SHH,SIX3,SLC19A3,SMC1A,STAG2,SUGCT,TG,TGIF1,TPO,TRHR,TSHB,TSHR,TSR2,YY1,ZIC2,MTRR,ATG7,SLC1A2,GCH1,TBK1,TWNK,DLD,KRT8,CYP24A1,KCNMA1,SLC4A1,ND1,ASS1,RRM2B,KCNJ11,KRT18,MMUT,NAA10,CDH23,CPT2,SIK1,SFRP2,CYP11B2,CPT1A,POLG2,H3-3A,HMBS,SLC25A20,ASPA,OTC,NDUFB11,NBAS,ATP13A2,SLC25A4,HADHA,FBP1,TCN2,NDUFS7,PDHA1,NDUFS3,NDUFS4,USP8,CPS1,ASL,PHIP,NDUFS1,SLC25A22,NDUFS2,USP18,ND2,SLC7A7,ND3,TES,NDE1,SLC22A5,NDUFV2,BNC2,GALT,SLAMF1,EIF2B5,NDUFV1,SLC25A13,GABRB2,HADHB,DPYS,HJV,ALDOB,ABAT,GLDC,SLC39A4,BTD,FOXRED1,NDUFS8,SLC19A2,NDUFA1,DBT,ACADM,NDUFAF2,PURA,MMACHC,EIF2B2,NDUFAF3,HLCS,HADH,TIMMDC1,SLC25A19,ACADVL,CACNB4,GK,EIF2B4,EIF2B1,ACADS,NUBPL,AMT,RMND1,ATP5F1D,NDUFAF5,MCCC2,PCCA,BCKDHB,NDUFS6,CA5A,NDUFAF4,GYS2,ACADSB,HMGCL,PCCB,BCKDHA,MMAB,NDUFB9,NDUFAF1,MCCC1,NDUFB3,NDUFA11,IVD,NDUFA6,SLC25A15,LMBRD1,TMEM126B,LYRM7,NAGS,NDUFB10,EIF2B3,TRNT,ACADL,MMAA,MMADHC,GCSH,BOLA3,ABCD4,NFU1,MRPS16,NAXD |
| Intermittent claudication | [HP:0004417](https://hpo.jax.org/app/browse/term/HP:0004417);  UMLS: C0021775 | CACNA1G-AS1,AGXT2,ANGPTL6,EIF3E,FURIN,DHX40,KLRB1,AGXT,XYLT1,XYLT2,BAK1,BAD,ALDH7A1,F8,ABCC6,BCAR1,BBC3,ZMPSTE24,FGF13,SIRT2,MPL,NLRP1 |
| Knee pain | HP: 0030839  UMLS: C0231749 | MARCHF6,PTCRA,MMD,CLEC11A,PCSK6,F11,ZNF410,FPR1,COL9A2,DPEP1,ACLY,BSCL2,GLA,GDF5,COMP,TACR1,B2M,COL2A1,COL9A1,COL9A3,LMX1B,MATN3,SLCO2A1,TONSL,TRAPPC2 |
| Exercise- induced myalgia | [HP:0003738](https://hpo.jax.org/app/browse/term/HP:0003738);  UMLS: C1850830 | CPT2,LPIN3,PHKA1,PGAM2,SLC25A42,AMPD3,RNASEH1,MYF6,MTMR14,ACADVL,PYGM,ACADM,MATR3,LPIN2,ANO5,LPIN1,TRIM32,AGPAT2,DYSF,AMPD1,FLNC,CAV3,CAVIN1,LAMA2,BIN1,BSCL2,PGK1,DNM2,RRM2B,FKRP,STIM1 |
| Limitation of joint mobility | HP: 0001376;  UMLS: C1857108 | ADCY6,TRAPPC2,MYH8,LGI4,CLCF1,ZNHIT3,CKAP2L,SLC25A19,PSTPIP1,ALOXE3,BHLHA9,ADAMTS10,GLE1,LMBR1,GBE1,ABCA12,ANKH,ALOX12B,CRLF1,CD96,KLHL7,SLCO2A1,TBX4,MVK,BMPR1B,TYROBP,CHRNG,ERF,LTBP2,SLC40A1,COL9A3,TGM1,MAFB,MYH3,ZC4H2,KIF1A,SCN4A,CNTNAP1,ERCC8,PLP1,CD247,ACVR1,PIEZO2,TREM2,HPGD,GDF5,ERCC6,COMP,USP9X |
| Fatigue | [HP:0012378](https://hpo.jax.org/app/browse/term/HP:0012378);  UMLS: C0015672 | ACTC1,AP2S1,ASXL1,BRCA1,BRCA2,CARMIL2,CBL,CD244,CIITA,CITED2,COL1A2,COL9A1,COL9A2,COL9A3,COMP,DMPK,DNMT3A,EIF2AK4,EPAS1,GATA4,GATA6,GRM1,IL18BP,IVNS1ABP,KCNE1,KCNN4,KCNQ1,KL,MC2R,MDM4,MORC2,MRAP,MYH6,NF1,NFKBIL1,NNT,PALB2,PALLD,PGM1,PIEZO1,PYGL,SCN4A,SLC22A12,SLC22A4,SLC2A9,SLC4A1,SMAD3,SMAD4,STAR,SVIL,TAFAZZIN,TBX20,TICAM1,TK2,TLL1,TLR3,TRAF3,TRHR,TXNRD2,UBA1,UNC93B1,YY1,RPS17,MMADHC,SLCO1C1,CCDC78,HELLPAR,POLDIP3,RPL27,IL36A,PNPLA8,APELA,RPL35,TEX10,TRIM55,CRCP,LMCD1,RAB11FIP1,LVRN,WDR18 |
| Myalgia | [HP:0003326](https://hpo.jax.org/app/browse/term/HP:0003326);  UMLS: C0231528 | ACADM,ACADVL,ACP5,ALMS1,AMPD3,BIN1,CHCHD10,COX6B1,DNM2,DYSF,FLNC,HLA-DRB1,HPDL,KY,MATR3,MDM4,MIEF2,MTMR14,MTTP,NOD2,PGAM2,PGK1,PHKA1,PHKA2,PHKG2,PYGM,RNASEH1,STING1,SVIL,TRIM32,RREB1,STIM1,PRTN3,LDHA,SERPINC1,NTN1,OPA1,PNPLA2,FKTN,FKRP,PDE5A,MYH7,TWNK,JMJD1C,ORAI1,AIF1,ND1,SLC4A1,EXT1,GP1BB,POMT1,RRM2B,HMGCR,ND5,CCR1,TRNL1,COL5A1,HIRA,UFD1,GLA,POLR3A,SLCO1B1,UCN,RYR2,ARVCF,ANK1,CPT2,SEC24C,SCN9A,ND6,ADA2,ND4,COX3,P2RX4,P2RX5,HLA-DPA1,LAMA2,SCN4A,TRNS1,ERAP1,CNBP,NLRC4,ANKRD11,MB,POLG2,CAVIN1,HMBS,PMAIP1,TRNW,C4A,CAV3,CAPN3,SEMA4D,P2RX3,CRPPA,FDXR,COL5A2,SLC25A4,NLRP12,HADHA,TNXB,OPN1MW,AMPD1,DNASE1L3,MVK,TRNS2,ROM1,TRNF,MSTO1,LEMD3,MAT1A,ZNF469,TK2,FABP3,BDKRB2,P2RX5-TAX1BP3,TRNQ,DGUOK,CLCNKB,SNRPB,TRNH,NLN,LPIN1,OPN1MW2,OPN1MW3,DMPK,TRNK,IGF2-AS,ANO5,HADHB,MAP2K3,PRDM5,DNA2,LPIN2,GSC,PIGT,IL12A-AS1,PIK3C2A,UBAC2,SPTA1,CLCN1,TRNE,TRAPPC11,SPTB,SLCO2B1,ASIC3,KLRC4,MYOT,DSE,TMEM126B,KCNA4,TRMU,CASQ1,MLX,ACAD9,BVES,KCNJ18,EPB42,LALBA,DOLK,RBCK1,ACADL,MORC3,OTULIN,CCDC78,ENO3,PYROXD1,DNAL4,FDX2,SH3BP5,PRLHR,TRI-AAT9-1,LILRB5 |
| Generalized morning stiffness | HP:0005197;  UMLS: C4025238 | PRG4,CACNA1S |
| Palpitations | HP:0001962;  UMLS: C0030252 | ABCC8,ACTC1,CDH2,CITED2,DNMT3A,EPAS1,GCGR,HNF1A,JUP,KCNJ11,KCNJ5,MYH6,NF1,NKX2-5,PNPLA2,TBX20,TLL1,UCP2,YY1,NPPA,SCN5A,FKRP,TWNK,KIF1B,SDHA,SDHC,KCNH2,RRM2B,CDH23,SCN9A,DAXX,BSCL2,MAX,CNBP,POLG2,CAVIN1,CAV3,SLC25A4,KCNJ2,NR2F2,PRKAG2,AGPAT2,CLCNKB,CACNA1S,MDH2,TTN-AS1,DSG2,MYL2,SDHAF2,PKP2,KCNA5,DSC2,KCND3,KCNE2,TMEM94,GABRA3,TMEM127,CACNA2D1,TMEM43,DLST,SLC25A11,CRELD1,ISCU,MYL3,CPA6,BVES,GYG1,KCNJ18,MYL4,JPH2,STRN4 |
| Headache | HP: 0002315;  UMLS: C0018681 | ACP5,ACSF3,ACVRL1,AKT1,ALX4,AMACR,AMER1,AP2S1,ATP1A2,BAP1,BCAT2,BMPR1A,C4A,CDKN1B,COL1A1,COL3A1,COL4A1,COL5A1,COL5A2,COX3,CPT2,CSNK1D,CYTB,DEPDC5,DLST,DNM1L,DNMT3A,ELANE,ENG,EPAS1,EPCAM,ESR1,F8,FAN1,FH,GABRG2,GATA2,GBA,GCDH,GDF2,GP1BA,GP1BB,GP9,GPR101,GRIN2A,HLA-B,HLA-DRB1,IL10,IL12B,IL23R,KCNK18,KDELR2,KIF1B,KL,KRAS,LGI1,MARCHF6,MAX,MBTPS2,MDH2,MLH1,MLH3,MSH2,MSH6,MYD88,MYORG,ND1,ND4,ND5,ND6,NDP,NFIX,NOP56,NOTCH3,OPA1,PDGFRB,PEX11B,PGK1,PMS1,PMS2,POLG2,PRKAR1A,PRRT2,RELA,RELN,RET,RNASEH1,RRM2B,SDHA,SDHAF2,SDHB,SDHC,SDHD,SEMA4A,SLC25A11,SLC25A4,SLC6A19,SMAD3,SMAD4,SMARCE1,SMO,SPOP,SRPX2,STARD7,STIM1,TBK1,TENT5A,TERT,TGFBR2,TICAM1,TLR3,TMEM127,TNFRSF1A,TRAF3,TRNC,TRNF,TRNK,TRNL1,TRNQ,TRNS1,TRNS2,TRNV,TRNW,TWNK,UNC93B1,YEATS2,HELLPAR,PLEKHA1,ATG13,SH3BP5,MRVI1,BTN2A2,ZFHX2,NOL4L |
| Hyperhidrosis/Hyperhidrosis disorder | HP: 0000975;  UMLS: C0020458 | ALDOB,ATP1A2,ATP1A3,BRAF,CACNA1A,CFTR,CLCN6,COL17A1,DLST,DNMT3A,DSG1,EPAS1,FH,GDF1,H19,HLA-DRB1,HNRNPK,IGF2,ITGB4,KDELR2,LAMA3,LAMB3,LAMC2,MBTPS2,MDH2,MED13L,NF1,NPM1,NTRK1,P4HA2,PERP,RSPO1,SDHA,SDHAF2,SLC1A3,SLC25A11,TENT5A,TRPV3,WNT10A,YY1,MPL,KRT5,THPO,KIF1B,HPGD,SDHC,AIP,LIFR,KCNJ11,KRT14,KRT17,MALT1,PSMB8,MAP2K2,BLM,DDC,CDH23,SCN9A,BSCL2,DKC1,DST,ASCL1,MAX,SLC18A2,NLRC4,CUL4B,KIF1A,CAVIN1,HMBS,SLURP1,MAGEL2,COL6A3,PHOX2B,KRT16,KCNA1,TINF2,PARN,KRT1,SLCO2A1,ELP1,AGPAT2,COL6A1,CLCNKB,CACNA1S,CRLF1,PLAA,COL6A2,NGLY1,CTC1,WRAP53,CDK13,EDN3,SRCAP,KRT6A,HINT1,RETREG1,NHP2,FUCA1,JUP,NOP10,SPR,GPR101,USB1,GABRA3,IGHMBP2,TMEM127,HEXB,SEPSECS,KRT6B,SUCLG1,SCN11A,CLCF1,GNA14,KRT9,MLX,KCNJ18,KLC2,SERPINB7,FLRT1,LONP2 |
| Shortness of breath | HP: 0002094;  UMLS: C0013404 | ABCA3,ABCC6,ACADM,ACADS,ACADVL,ACTA2,ACTC1,ADAMTS13,ADCY6,ADNP,AGRN,AIFM1,AIMP2,ALMS1,ASAH1,ASXL1,ATP6,B3GALT6,BCOR,BMPER,BTD,CBL,CDC45,CDC6,CDT1,CHAT,CHRNA1,CHRNB1,CHRNE,CITED2,CLCNKB,CNTNAP1,COA8,COL1A2,COL2A1,COL4A5,COL4A6,COLQ,COPA,COQ7,COX7B,CPT2,CREBBP,CRELD1,CYB5A,CYB5R3,DHX16,DISC1,DMPK,DNA2,DNAAF3,DNAJB6,DNAJC21,DOK7,DPM1,DPM2,DSP,DYNC2LI1,EDA,EFTUD2,ELN,ENPP1,EP300,EPHB4,EPOR,ERF,ETFA,ETFB,ETFDH,FAM20C,FBN1,FGFR1,FGFR2,FOXE3,FOXF1,FOXP3,GALC,GATA4,GATA6,GMNN,GNAI3,GTPBP3,GYG1,HCCS,HLCS,IFT52,IFT81,IL1RN,IRF2BP2,ITGA3,JAK2,KAT6A,KCNA1,KLHL7,KRT16,KRT17,KRT6A,KRT6B,LAMB2,LGI4,LIFR,LMNA,LOX,LRP4,LYRM4,MAPT,MEGF10,MFAP5,MMAA,MMAB,MMACHC,MMUT,MPC1,MUC5B,MUSK,MYH11,MYH6,MYL3,MYLK,MYO9A,MYPN,NAGS,ND1,ND2,ND4,ND5,ND6,NDUFAF3,NDUFB11,NDUFB8,NDUFS2,NEB,NEMF,NGLY1,NKX2-1,NPM1,NR2F2,NUMA1,NUP214,ORC1,ORC4,OTX2,PARN,PGM1,PLCB4,PLEC,PMM2,POLG,POLG2,POMT1,PRKAR1A,PRKG1,PRRX1,PSAP,PUF60,PURA,PYGM,RAPSN,RARA,RELN,RNF13,RPS26,RPS28,RRM2B,RTEL1,RUNX1,RUNX2,SBDS,SDCCAG8,SERPING1,SETBP1,SFTPA2,SFTPC,SH2B3,SIK1,SLC12A3,SLC18A3,SLC25A3,SLC25A4,SLC2A10,SLC35A1,SLC52A3,SLC5A7,SMAD3,SMAD4,SNAP25,SNRPN,SOX9,SRP54,SSR4,STAT3,STAT5B,STN1,STT3B,SURF1,SYT2,TBC1D24,TBL1XR1,TBX20,TERC,TERT,TFG,TGFB2,TGFB3,TGFBR1,TGFBR2,TK2,TLL1,TNNI3,TNNT2,TRAK1,TRIP11,TRMT5,TRMU,TRNL1,TRNN,TRNS1,TRNV,TRNW,TRPV6,TUBB4A,TWNK,UBE3B,USP9X,VAMP1,VPS33A,XYLT1,ZBTB16,ZFPM2,ZMPSTE24,DNAH7,MRPL3,STRN4,PDA1 |
| Muscle tenderness | UMLS: C0240419 | CCL8 |
| Low Limb pain/Pain in lower limb | [HP:0012514](https://hpo.jax.org/app/browse/term/HP:0012514);  UMLS: C0023222 | ACP5,B2M,BSCL2,COL2A1,COL9A1,COL9A2,COL9A3,COMP,KRT14,KRT16,KRT17,KRT5,KRT6A,KRT6B,MATN3,MFN2,NLRP12,RASA1,LBP,ATP6,PHEX,ALDH18A1,ZC4H2,RSS,PRKRA,TRNK,CNTN3,MOCOS,SMCP,ZNF35,MYOT,SPNS1,NARS1,CBX1,ERLIN1,BLOC1S2 |
| Leg muscle stiffness | HP: 0008969;  UMLS: C4024610 | ABCD1,ATP6,PODXL,KIF1A,ATP13A2,SYNJ1,SPAST,KIF5A,VAMP1,ZFYVE26,DARS2,DNAJC6,LDB3,ANO10 |
| Difficulty walking | HP: 0002355;  UMLS: C0311394 | ABCD1,ACOX1,AKT1,ALAD,ALS2,BAP1,BSCL2,BVES,CAPN1,CDK19,CDK8,CDKL5,CELF2,COL12A1,COL6A1,COL6A3,DALRD3,DDX6,DLAT,DNM1L,DYM,EIF2AK3,ERCC1,ERCC4,ERCC6,ERCC8,FGF13,FOXG1,FTL,FUS,FUZ,GABRA2,GABRA5,GALC,GIPC1,HERC1,HTT,INTS1,KCNJ10,MARCHF6,MATN3,MECP2,MED25,MIEF2,MPZ,MTRFR,MYO1H,NTRK1,PIK3CA,PKP1,PLP1,PMP22,POLG2,PUS3,PYCR2,RSPRY1,SIGMAR1,SLC7A6OS,SMARCE1,SMO,SORD,SQSTM1,TERT,TMEM251,TRAF7,TRPV4,UBAP1,WARS2,ADAR,USP9X,CYP27B1,SCN8A,GRIN2B,SLC1A2,PNPLA2,FKTN,FKRP,GJB1,CRYAB,MYH7,TREX1,SCN2A,ORAI1,CHAT,POMT1,ASAH1,ATP7B,TPM2,STXBP1,PSAP,FHL1,COL5A1,CYP27A1,ACTA1,ATP6,DNM2,HK1,STUB1,POMT2,PEX6,SAMHD1,GABRG2,BIN1,COASY,TPP1,SLC25A1,EEF1A2,CLTC,CYP2R1,TRNW,CAPN3,HCN1,CRPPA,ATP13A2,PPP3CA,CCN6,FLNC,LRP4,SETX,HSPB8,WASHC5,FA2H,SYNJ1,SPG11,SURF1,SLC30A10,GMPPB,SPAST,ATP6V1A,TAF1,MSTO1,DYSF,UBA5,SYNGAP1,TK2,SPART,RNASEH2C,RNASEH2A,GJC2,RNASEH2B,TIA1,DNM1,MPV17,SLC5A7,COL6A2,HACE1,RARS1,EIF2S3,SACS,SLC18A3,COL13A1,NEB,SLC13A5,NACC1,MYPN,KCNA2,GFPT1,BICD2,SCN3A,ADCY5,CIZ1,SBF2,FGF14,FGF12,ANO5,NUS1,KCND3,GABRB2,YWHAG,DPAGT1,CHCHD10,DNA2,MORC2,AGRN,VAMP1,WDR48,CNKSR2,GLE1,MATR3,AP4B1,CYFIP2,GBA2,IGHMBP2,NDUFS8,ELOVL5,DARS2,AP4M1,MICU1,SGCD,ABHD5,C19orf12,TGM6,PARS2,B4GALNT1,GPAA1,AARS1,ERLIN2,SYT2,MYO9A,TRAPPC11,PRX,GPT2,CACNA1B,WARS1,PGAP1,KCNB1,GRIN2D,SLC25A19,REEP1,GOSR2,ARL6IP1,SGCB,ACTL6B,ARSI,AP4E1,NOP56,NPHP3-ACAD11,SH3TC2,IBA57,ALG2,NUBPL,SZT2,GRID2,MTMR14,PLEKHG5,GAN,TRAK1,AP4S1,PDK3,C12orf65,TCAP,TTBK2,KLHL41,ARV1,RAB11B,AP3B2,CPT1C,MYF6,NECAP1,SPG21,NKX6-2,MYOT,SLC34A3,KCNC3,RTN2,DHTKD1,STRADA,DDHD1,ALG14,GYG1,TBC1D23,KBTBD13,KLC2,ERLIN1,SLC30A9,KY,LRP12,HPCA,PMP2,PYROXD1,FDX2,FLRT1,FBXO38,RNU12,KLHL9 |
| Lower limb muscle weakness | HP: 0007340  UMLS: C1836296 | ABCD1,ADAR,ADSS1,AFG3L2,AGRN,AKT1,ALDH18A1,ANO5,AP5Z1,ASAH1,ATL1,ATP6,BAP1,BSCL2,CACNA1S,CAPN1,CAPN3,CAV1,CHAT,CHCHD10,COL13A1,CPT1C,CYP7B1,DCTN1,DDHD1,DDHD2,DES,DGUOK,DSTYK,DYSF,ERLIN2,EXTL3,FAM126A,FBXO38,FGD4,FHL1,FLI1,FLNC,GALC,GARS1,GBA2,GJB1,GMPPB,GNE,HADHA,HADHB,HARS1,HINT1,HK1,HSPB1,HSPB3,HSPB8,HSPD1,IBA57,KCNJ10,KCNJ18,KIF1A,KIF5A,L1CAM,LMNA,MAPT,MFN2,MME,MORC2,MPZ,MTHFR,MTRFR,MTTP,MYH14,MYH7,MYO9A,MYPN,NDRG1,NEFH,NEFL,NF1,NF2,NIPA1,NUP62,PDGFB,PDK3,PIEZO2,PIK3CA,PLEC,PLP1,PMP22,PNKP,PNPLA6,PRNP,RASA1,REEP1,RTN2,SARDH,SCO2,SCYL1,SLC18A3,SLC25A1,SLC33A1,SLC5A7,SMARCB1,SMARCE1,SMO,SNAP25,SORD,SPART,SPAST,SPG11,SPG21,SPG7,SUFU,SYNE1,SYT2,TCAP,TDP1,TERT,TFG,TPM2,TPM3,TRPV4,TTN,VAMP1,VCP,VHL,WASHC5,ZFYVE26,ZFYVE27,MPV17,MYOT,DYNC1H1,IGHMBP2 |
| Memory impairment | HP: 0002354;  UMLS: C0233794 | MYORG,REEP2,ACSF3,EIF2B3,EIF2B1,EIF2B4,EIF2B2,ABCA7,SLC20A2,VAMP1,FGF14,SLC25A13,PRKCG,EIF2B5,FAN1,GNE,JPH3,CAMTA1,TMEM106B,TRNQ,AFG3L2,MLH3,TRNF,TRNS2,SPAST,ECM1,TYROBP,PMS1,SEMA4A,TRNW,C4A,TRNS1,ALDH18A1,PSEN2,HTT,COX3,ND4,MOG,ADA2,STUB1,ND6,CHMP2B,TRNL1,ATXN1,PSAP,ND5,ND1,BMPR1A,GRIN2A,ATXN3,TREM2,PAH,TWNK,HCRT |
| Insomnia/ Sleeplessness/ Sleep onset insomnia /Difficulty sleeping | HP: 0002360,  [0031354](https://hpo.jax.org/app/browse/term/HP:0031354),  0100785;  UMLS:C0037317, C0235162, C0917801,  C4531177 | RLS3,STAC,IGSF6,FREM3,PRLHR,MARCHF11,FBXO3,NAGS,QARS1,SIAE,RLS1,PPP2R3C,PLEKHM2,HCRTR2,MDD1,AMT,DDAH2,TMED9,ARSI,VPS13C,MADD,DNAJC6,ZNF365,FSHMD1A,HRES1,PSG5,GSC,MAP3K10,UNC80,CD200R1,HCRTR1,SMCP,SLC25A13,MOCOS,FARS2,PIGR,EBPL,IARS1,DDAH1,UBE2Z,DCTN1,CLCNKB,OAT,IS1,USE1,MTNR1A,TST,MPST,MTNR1B,NDUFS4,CRY1,MSTO1,PDC,MEIS1,ROM1,NDUFS7,ADRA2A,MAP6,AMPD1,TPPP,PPARGC1B,LMX1B,PKD2L1,SEMA4D,UTS2,MXD1,ZC4H2,HMBS,PER3,GTF2H5,OPRK1,TPH1,SIN3A,PODXL,WASF1,GABRB3,CENPJ,HTRA2,POTEKP,POTEM,ACTBL2,TBL2,PARK7,TIMELESS,REM1,ACOT7,BAZ1B,FHL5,CLIP2,CHDH,CDK5R1,TAM,ADORA2A,GTF2IRD1,RFC2,OPN1SW,PINK1,STAR,LRRK2,ARNTL,OCLN,CYP2C9,ACTG2,CTCF,HCRT,GTF2I,ABCB11,ABCB4,ATP8B1,CPOX,ELN,GALNT2,HLA-DQB1,HLA-DRB1,HTT,MEN1,NCF1,NR1H4,PDE2A,PRKN,PRNP,SLC12A3,SNCA,SYNJ1,TUBB3,UCHL1,YY1,CSN1S1,IGHG1,CHD8,PMCH,CNTN3,HOPX,ST13,STIP1,SUCLA2,KCNJ11,PSG3,IGLON5,FIGNL1,IRAK1BP1,PTLS,RBM12,SLITRK1,STOX1,DHX30,ZNF687,MINPP1,AP2S1,SLC34A3,NUMA1,IRF2BP2,SNX10,LPIN2,SCARB2,AGXT,CLCN5,HABP2,CLCN7,LEMD3,SLCO2A1,SERPINF2,TYROBP,FOXE1,TCIRG1,CYP2R1,DMP1,PHEX,ZBTB16,PTH1R,CDC73,TRPS1,TBL1XR1,PSMB8,ATP7B,EXT1,TREM2,HPGD,KIF1B,CA2,CYP27B1,GBA,NABP1,HEPH,NRTN,ARL6IP1,BDNF-AS,VSNL1,SETD7,OPRL1,CAVIN2,PNOC,LGMN,EXT2,FIP1L1,NOL3,P2RX3,ATHS,SLC16A4,P2RX4,PDLIM3,S1PR1,ATRNL1,DLG3,SLC16A3,HCLS1,NAT10,SIGLEC7,WNT3A,PIEZO2,KHSRP,CXCL11,MEF2C,CBLIF,CCL27,TACR1,ASXL1,BCOR,CBL,CCND1,CTSK,CYP19A1,EHHADH,ELANE,ENPP1,FGF23,GATM,GNAS,IDH1,IDH2,KDELR2,KIT,MBTPS2,NDUFAF6,NOTCH2,NPM1,POLE,PRKAR1A,PSAP,PTPN11,RARA,RUNX1,SLC34A1,SQSTM1,STAT3,STAT5B,TEK,TENT5A,TET2,TGFB1,TNFRSF11A,TNFRSF1A,TNFSF11,VCP,VDR,WNT1 |
| Night sweats | [HP:0030166](https://hpo.jax.org/app/browse/term/HP:0030166);  UMLS: C0028081 | UBA1,SLC25A13,IFNGR1 |
| Heat intolerance | HP: 0002046 | CLDN10,EDA,EDAR,EDARADD,HNRNPK,ITPR2,KRT14,KRT5,LDHA,MAP2K1,MAP2K2,MBTPS2,NECTIN4,NFKBIA,OCA2,ORAI1,POFUT1,POGLUT1,PSENEN,SHANK3,UBE3A |
| Low back pain | [HP:0003419](https://hpo.jax.org/app/browse/term/HP:0003419);  UMLS: C0024031 | SERT,NET,DISC2,SLC6A4,LCS1,TSKU,NUBP1,RHAG,CISD3,EMSLR,BMP3,ZNF35,RTN1,TBX6,SPECC1,PES1,ITLN1,DNAJC5,ZFYVE9,SPEN,NAB2,MCF2L2,RTN4,FURIN,ADAMTS5,ADAMTS4,ADAMTS3,SPAST,HSPB8,PACC1,RGS6,ABCD1,ACP5,ALDH18A1,COASY,F11R,MARCHF1,HTRA1,ACAN,LBP,RARRES2,FST,GDF5,SEMA3A,IL6,IL4,SELL,PTGS2,IL10,CXCL8,TNF,POMC,CCL3,VEGFA,MPO,IL1B,UGT1A10,SLC22A9,UGT2B7,CX3CL1,HPGD,FAS,NOS2,PTGS1,NFKB1,UGT1A1,UGT1A9,FOS,CASP3,CYP1A2,CYP2C9,CCL2,ICAM1,MMP13,CALCA,NR3C1,ALB,CXCR4,BCL2,EGR1,UGT1A3,UGT1A8,CLU,IL1A,SLC22A7,GFAP,RELA |

Table S6 Network analysis of non-overlapping DEGs in early-stage hubs

| Gene | Average shortest path length | Fold change | *P* value | Co-expression correlation (P<0.05) |
| --- | --- | --- | --- | --- |
| TIAM1 | 1.00 ^a^ | 2.05096714 ↑ ^b^ | 0.0004956 ^a^ | √ |
| CD4 | 2.60 ^a^ | 1.595102823 ↑ | 0.0010162 ^a^ | √ |
| PLCG1 | 2.79  ^a^ | 1.836081726 ↑ ^b^ | 0.0014660 | √ |
| PRKCA | 2.95 ^a^ | 1.613305888 ↑ ^b^ | 0.0015110 | √ |
| CD28 | 3.02 ^a^ | 1.570784125 ↑ | 0.0004820 ^a^ | √ |
| PIK3CD | 3.03 ^a^ | 1.865419887 ↑ ^b^ | 0.0007645 ^a^ | √ |
| SERPINE1 | 3.12 | 0.602077797 ↓ | 0.0353637 |  |
| FOXO4 | 3.34 | 0.564929116 ↓ | 0.0242564 |  |
| GNAQ | 3.63 | 1.525491211 ↑ | 0.0300516 | √ |
| PAK2 | 3.86 | 1.743079232 ↑ ^b^ | 0.0011703 ^a^ | √ |
| E2F1 | 4.34 | 0.455630144 ↓ | 0.0462772 |  |
| MAP3K5 | 5.20 | 1.804781116 ↑ ^b^ | 0.0012219 ^a^ | √ |

^a^ Lower than the median, ^b^ higher than the median; ↑ up-regulation, ↓ down-regulation.

√ correlation analysis *P*＜0.05.

Median (average shortest path length)=3.299; median (FC)=1.604; median (P value)=0.001.

Table S7 Network analysis of non-overlapping DEGs in mid-stage hubs

| Gene | Average shortest path length | Fold change | *P* value | Co-expression correlation (P<0.05) |
| --- | --- | --- | --- | --- |
| HMOX1 | 1.00 ^a^ | 1.57079045 ↑ | 0.0023076 ^a^ | √ |
| RAC1 | 2.19 ^a^ | 1.561201499 ↑ | 0.0004335 ^a^ | √ |
| YWHAZ | 2.25 ^a^ | 2.005134074 ↑ | 0.0021571 ^a^ | √ |
| RHOA | 2.38 ^a^ | 1.500350215 ↑ | 0.0117903 | √ |
| XIAP | 2.41^a^ | 1.579514185 ↑ | 0.0025674 ^a^ | √ |
| TRAF6 | 2.52 ^a^ | 1.519820499 ↑ | 0.0003466 ^a^ | √ |
| PLK1 | 2.58 ^a^ | 0.656151702 ↓ | 0.0064213 | √ |
| PDGFRB | 2.58 ^a^ | 0.645206079 ↓ | 0.0074614 | √ |
| PIK3CB | 2.63 ^a^ | 1.569529881 ↑ | 0.0004899 ^a^ | √ |
| CHEK1 | 2.64 ^a^ | 0.57623882 ↓ | 0.0273798 | √ |
| STK11 | 2.66 ^a^ | 0.40822767 ↓ | 0.0001288 ^a^ | √ |
| HDAC4 | 2.72 ^a^ | 1.646992988 ↑ ^b^ | 0.0004086 ^a^ | √ |
| VCL | 2.79 ^a^ | 1.502972761 ↑ | 0.0021941 ^a^ | √ |
| IQGAP1 | 2.80 ^a^ | 2.30589492 ↑ ^b^ | 0.0000841 ^a^ | √ |
| RACGAP1 | 2.86 ^a^ | 0.634265161 ↓ | 0.0174277 | √ |
| CASP8 | 2.89 ^a^ | 1.6610604 ↑ ^b^ | 0.0087994 |  |
| GATA3 | 2.90 ^a^ | 1.795843011 ↑ ^b^ | 0.0001343 ^a^ | √ |
| AURKA | 2.92 ^a^ | 0.632722633 ↓ | 0.010799 | √ |
| BIRC5 | 2.93 | 0.540543442 ↓ | 0.0045923 | √ |
| BIRC2 | 2.93 | 0.618514342 ↓ | 0.0189262 | √ |
| ETS1 | 3.00 | 1.633364146 ↑ ^b^ | 0.0033717 | √ |
| ITGAM | 3.02 | 1.630391753 ↑ ^b^ | 0.0183465 | √ |
| POLR2A | 3.05 | 1.840855128 ↑ ^b^ | 0.0000545 ^a^ | √ |
| STAT6 | 3.08 | 1.867610408 ↑ ^b^ | 0.0005154 ^a^ | √ |
| MAPK12 | 3.08 | 0.649800658 ↓ | 0.0005588 ^a^ | √ |
| ACTN4 | 3.11 | 1.635091529 ↑ ^b^ | 0.0001402 ^a^ | √ |
| ICAM1 | 3.14 | 1.578026649 ↑ | 0.0135677 | √ |
| GNG2 | 3.14 | 1.527180881 ↑ | 0.0112263 | √ |
| CSNK1D | 3.22 | 1.64823268 ↑ ^b^ | 0.0006457 ^a^ | √ |
| TCF7L2 | 3.24 | 1.758004741 ↑ ^b^ | 0.0060334 | √ |
| CEBPA | 3.27 | 1.721500615 ↑ ^b^ | 0.0150812 | √ |
| PSEN1 | 3.32 | 1.839903711 ↑ ^b^ | 0.0002851 ^a^ | √ |
| BCL6 | 3.34 | 2.035606337 ↑ ^b^ | 0.0046772 | √ |
| TUBA1A | 3.37 | 2.206242671 ↑ ^b^ | 0.0025865 ^a^ | √ |
| SELL | 3.42 | 1.839370642 ↑ ^b^ | 0.0026222 | √ |
| RBPJ | 3.51 | 1.755658987 ↑ ^b^ | 0.0043899 | √ |

^a^ Lower than the median, ^b^ higher than the median; ↑ up-regulation, ↓ down-regulation.

√ correlation analysis *P*＜0.05.

Median (average shortest path length)=3.109; median (FC)=1.605; median (P value)=0.003.

Table S8 Network analysis of non-overlapping DEGs in late-stage hubs

| Gene | Average shortest path length | Fold change | *P* value | Co-expression correlation (P<0.05) |
| --- | --- | --- | --- | --- |
| APP | 2.37 ^a^ | 1.732928998 ↑ | 0.0052349 | √ |
| NFKB1 | 2.55 ^a^ | 1.536524045 ↑ | 0.0002676 ^a^ |  |
| ERBB2 | 2.62 ^a^ | 0.642238265 ↓ | 0.0039411 ^a^ | √ |
| RIPK1 | 2.71 ^a^ | 1.502772544 ↑ | 0.0032453 ^a^ |  |
| STAT1 | 2.77 ^a^ | 1.812529812 ↑ ^b^ | 0.0158178 | √ |
| CXCR4 | 3.09 ^a^ | 1.822823571 ↑ ^b^ | 0.0247879 | √ |
| AGT | 3.38 ^a^ | 0.665602623 ↓ | 0.0012120 ^a^ | √ |
| IRF7 | 3.41 | 1.832754607 ↑ ^b^ | 0.0122496 | √ |
| STAT2 | 3.46 | 2.072045944 ↑ ^b^ | 0.0039731 ^a^ | √ |
| HLA-A | 3.47 | 2.257051714 ↑ ^b^ | 0.0060570 | √ |
| LPAR1 | 3.51 | 2.067334224 ↑ ^b^ | 0.0002782 ^a^ | √ |
| IRF5 | 3.72 | 1.817887418 ↑ ^b^ | 0.0407868 |  |
| IRF2 | 3.75 | 1.625836633 ↑ | 0.0018066 ^a^ | √ |
| RHOB | 3.78 | 1.579047166 ↑ | 0.0264066 | √ |
| SLC2A4 | 3.79 | 0.584692179 ↓ | 0.0457033 |  |

^a^ Lower than the median, ^b^ higher than the median; ↑ up-regulation, ↓ down-regulation.

√ correlation analysis *P*＜0.05.

Median (average shortest path length)=3.545; median (FC)=1.733; median (P value)=0.005.


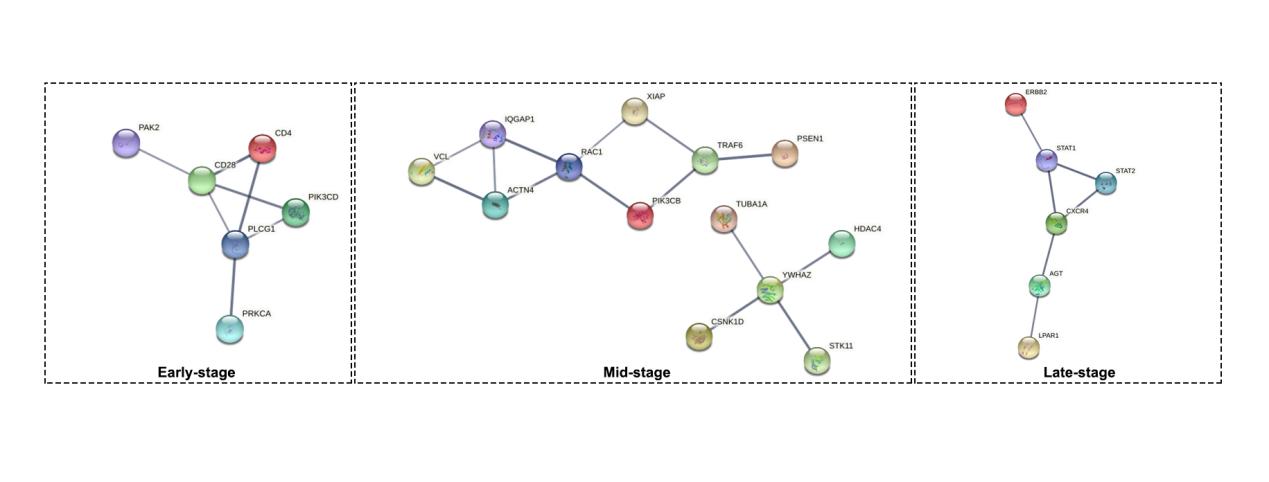


Figure S1 PPI network of selected genes following the network screening criteria

Table S9 Primer list of candidate biomarkers

| Stage | Gene | Primer sequence（5’-3’） | Base number |
| --- | --- | --- | --- |
| Early-stage | PAK2 | 1. GGCTGTGCTGGATGTCCTAAAG 2. GTCACTACTGCGGGTGCTT | 22  19 |
|  | CD28 | F- TCAGGACAAAGATGCTCAGGC  R- GACAAAGGTGTTTCCCTGTTACT | 21  23 |
|  | CD4 | F- ATCCAAGCTGAATGATCGC  R- GTCAATCCGAACACTAGCAA | 19  20 |
|  | PIK3CD | F- CTGGCTGAAGTCCAAGAACC  R- TGAAATTCCCCAGAAAGTGG | 20  20 |
|  | PLCG1 | F- GACAGAGTTTGTGGTGGACA  R- ACTGGGAAAGTAGCCTGAGC | 20  20 |
|  | PRKCA | F- GAACAGGGAGATCCAGCCAC  R- GTTAAGACGGGCTGTCCTCG | 20  20 |
| Mid-stage | VCL | F- GCCAAGCAGTGCACAGATAA  R- GCCTGCTCAGACTCCTCATC | 20  20 |
|  | IQGAP1 | F- TCTACTGTATCCATGCACTC  R- CTGACAGTTCATTAGCCAAG | 20  20 |
|  | ACTN4 | F- CCTTAGGTTCGCCATCCAGG  R- AGAGTGCCCACAATATCCTCTG | 20  22 |
|  | RAC1 | F- TTGAAAATGTCCGTGCAAA  R- GATCGTGTCTTTATCATCCCT | 19  21 |
|  | PLCG1 | F- GACAGAGTTTGTGGTGGACA  R- ACTGGGAAAGTAGCCTGAGC | 20  20 |
|  | XIAP | F- CAGCATCAACACTGGCACGAG  R- GCATGACAACTAAAGCACCGC | 21  21 |
|  | PIK3CB | F- GGAACGCCAGGCAGTGTATGT  R- ATAACCACGGGGCCCTAAG | 21  19 |
|  | TRAF6 | F- GATCTAGACTGCCCTACAGCC  R- CAGCTCCCGGATTTGATGGT | 21  20 |
|  | PSEN1 | F- TATACCCGGAAGGATGGGCA  R- GCCAGGCATGGATGACCTTA | 20  20 |
|  | TUBA1A | F- TCTGCTGAGAAAGCCTACCAT  R- GACATCTTTGGGAACCACGTCA | 21  22 |
|  | HDAC4 | F- GGGCTTTTGCTACTTCAACTCC  R- GATCTTGCTCACGCTCAACC | 22  20 |
|  | YWHAZ | F- GAGCAGGCTGAGCGATATG  R- ACCTACGGGCTCCTACAACA | 19  20 |
|  | STK11 | F- GCTCTTACGGCAAGGTGAAG  R- TTTTGTGCCGTAACCTCCTC | 20  20 |
|  | CSNK1D | F- CGTCAACATCTCCTCGTCCG  R- CCATGTCCCTGCTCCTCTGT | 20  20 |
| Late-stage | STAT2 | F- ATCCAGGCCAAAGGGAAGAC  R- GCATCCAGCACCTCCTTTCT | 20  20 |
|  | STAT1 | F- GTGAAGTTGAGAGATGTGAATGAG  R- TGGAGATCACCACAACGGG | 24  19 |
|  | ERBB2 | F- GTCACCTACAACACAGACACGTT  R- TGCCCAGACCATAGCACACTCG | 23  22 |
|  | CXCR4 | F- CTGCCTTACTACATTGGGAT  R- GTGCACAGTGTTCTCAAACTCAC | 20  23 |
|  | AGT | F- CAAAGACCCCACCTTCATACCTG  R- CCCTCAACTTGTCTTCGGTGT | 23  21 |
|  | LPAR1 | F- TCACATCTTTGGCTATGTTCG  R- TCATGGTATCCCGATTCCG | 21  19 |

F: Forward primer. R: Reverse primer.

Table S10 Relative gene expression level of the candidate biomarkers from RT-PCR analysis base on the validation cohort

| Stage | Gene | 2^(-△△Ct) * | | |
| --- | --- | --- | --- | --- |
|  |  | Non-NONFH control group | NONFH group | *P* |
| Early-stage | PRKCA | 1.00±0.69 | 2.44±1.07 | 0.001* |
|  | PLCG1 | 1.00±1.01 | 1.34±0.54 | 0.408 |
|  | PIK3CD | 1.00±0.57 | 1.62±0.62 | 0.031* |
|  | CD28 | 1.00±1.03 | 1.02±0.48 | 0.661 |
|  | CD4 | 1.00±0.71 | 6.29±2.74 | 0.000* |
|  | PAK2 | 1.00±0.60 | 1.02±0.42 | 0.604 |
| Mid-stage | HDAC4 ^#^ | 1.00±0.88 | 0.02±0.06 | 0.006* |
|  | IQGAP1 | 1.00±0.61 | 2.21±1.21 | 0.003* |
|  | YWHAZ | 1.00±0.64 | 1.22±0.48 | 0.411 |
|  | STK11 | 1.00±0.49 | 1.15±0.37 | 0.455 |
|  | TUBA1A | 1.00±0.90 | 2.10±0.89 | 0.005* |
|  | CSNK1D | 1.00±0.80 | 0.44±0.20 | 0.058 |
|  | PSEN1 | 1.00±0.71 | 1.05±0.50 | 0.605 |
|  | VCL | 1.00±1.02 | 2.80±2.16 | 0.002* |
|  | PIK3CB ^#^ | 1.00±0.55 | 0.45±0.24 | 0.017* |
|  | ACTN4 | 1.00±0.71 | 0.74±0.45 | 0.363 |
|  | TRAF6 | 1.00±0.72 | 0.61±0.15 | 0.123 |
|  | RAC1 | 1.00±0.71 | 1.31±0.39 | 0.255 |
|  | XIAP | 1.00±0.79 | 0.75±0.32 | 0.371 |
| Late-stage | STAT1 | 1.00±1.10 | 1.09±0.73 | 0.834 |
|  | STAT2 | 1±0.75 | 2.62±1.38 | 0.008* |
|  | ERBB2 | 1.00±1.28 | 0.84±0.42 | 0.297 |
|  | CXCR4 | 1.00±0.88 | 2.35±1.6 | 0.025* |
|  | AGT | 1.00±0.81 | 1.94±1.93 | 0.662 |
|  | LPAR1 | 1.00±0.98 | 2.13±0.74 | 0.013* |

* Normalization of the original data using the non-NONFH control group in gene expression, GRAPH was set as the internal control.

^#^ The validation result was contrary to the gene expression microarray data.

Table S11 Prediction features of NONFH candidate gene biomarkers

| Comparison | vs. NC group | | | | | |  | | vs. N&S group | | | | | |
| --- | --- | --- | --- | --- | --- | --- | --- | --- | --- | --- | --- | --- | --- | --- |
| Feature | Specificity | Sensitivity | Accuracy | Precision | F1 |  | | Specificity | | Sensitivity | Accuracy | Precision | F1 |  |
| PRKCA | 88.89% | 84.21% | 85.71% | 94.12% | 88.89% |  | | 52.78% | | 84.21% | 63.64% | 48.48% | 61.54% |  |
| PIK3CD | 40.00% | 100.00% | 80.00% | 76.92% | 86.96% |  | | 56.10% | | 85.00% | 65.57% | 48.57% | 61.82% |  |
| CD4 | 100.00% | 82.35% | 88.00% | 100.00% | 90.32% |  | | 65.71% | | 100.00% | 77.78% | 61.29% | 76.00% |  |
| IQGAP1 | 90.00% | 68.75% | 76.92% | 91.67% | 78.57% |  | | 15.00% | | 100.00% | 39.29% | 32.00% | 48.48% |  |
| TUBA1A | 70.00% | 100.00% | 88.89% | 85.00% | 91.89% |  | | 75.61% | | 70.59% | 74.14% | 54.55% | 61.54% |  |
| VCL | 100.00% | 82.35% | 88.00% | 100.00% | 90.32% |  | | 53.85% | | 76.47% | 60.71% | 41.94% | 54.17% |  |
| STAT2 | 75.00% | 92.31% | 85.71% | 85.71% | 88.89% |  | | 48.84% | | 92.31% | 58.93% | 35.29% | 51.06% |  |
| CXCR4 | 66.67% | 90.91% | 78.26% | 71.43% | 80.00% |  | | 50.98% | | 90.91% | 58.06% | 28.57% | 43.48% |  |
| LPAR1 | 60.00% | 100.00% | 80.95% | 73.33% | 84.62% |  | | 80.00% | | 100.00% | 85.37% | 64.71% | 78.57% |  |

NC group: Non-NONFH control group. N&S group: Non-NONFH & other stage group
